# Risk Factors, Diagnosis and Management of Bone Stress Injuries in Adolescent Athletes: A Narrative Review

**DOI:** 10.3390/sports9040052

**Published:** 2021-04-16

**Authors:** Belinda Beck, Louise Drysdale

**Affiliations:** 1 School of Health Sciences and Social Work, Griffith University, Gold Coast, QLD 4222, Australia; louise.drysdale2@griffithuni.edu.au; 2 The Bone Clinic, Brisbane, QLD 4151, Australia; 3 Queensland Ballet, Brisbane, QLD 4101, Australia

**Keywords:** bone stress injury, adolescent, athletes, stress fracture, injury management, risk factors

## Abstract

Physical activity is known to be beneficial for bone; however, some athletes who train intensely are at risk of bone stress injury (BSI). Incidence in adolescent athlete populations is between 3.9 and 19% with recurrence rates as high as 21%. Participation in physical training can be highly skeletally demanding, particularly during periods of rapid growth in adolescence, and when competition and training demands are heaviest. Sports involving running and jumping are associated with a higher incidence of BSI and some athletes appear to be more susceptible than others. Maintaining a very lean physique in aesthetic sports (gymnastics, figure skating and ballet) or a prolonged negative energy balance in extreme endurance events (long distance running and triathlon) may compound the risk of BSI with repetitive mechanical loading of bone, due to the additional negative effects of hormonal disturbances. The following review presents a summary of the epidemiology of BSI in the adolescent athlete, risk factors for BSI (physical and behavioural characteristics, energy balance and hormone disruption, growth velocity, sport-specific risk, training load, etc.), prevention and management strategies.

## 1. Introduction

Bone stress injuries (BSI) are typically associated with athletic or occupational overuse loading of the skeleton. The primary negative consequence of bone stress injuries (BSI) is time loss from training and competition which, at the elite junior level (specifically, under age 20), may have profound implications for professional opportunities. More broadly, BSIs represent a public health concern from the perspective that they may hinder ongoing participation in physical activity. While numerous factors have been associated with the incidence of BSI, the extent to which each factor contributes to the development of stress fractures in the young athlete is yet to be fully understood. The following is a consolidation of the literature around stress fractures and BSI in adolescent athletes, including epidemiology, risk factors, diagnosis, management and prevention, with a focus on the highest quality evidence. 

## 2. Defining Stress Fracture and Bone Stress Injury (BSI)

Stress fractures are focal structural weaknesses in bone occurring in response to the repeated application of stresses below the fracture threshold. The term bone stress injury (BSI) encompasses a range of bone tissue disturbances resulting from prolonged repetitive loading ranging from periostitis (inflammation of the periosteum), to periosteal, endosteal and bone tissue oedema (inflammation of the bony linings, or within the cortical bone tissue itself), to partial or complete stress fracture. All may be referred to as bone stress reactions or bone stress injuries; however, stress reaction typically denotes a less severe stage than stress fracture [1,2].

As bone is well supplied with nociceptors, BSIs tend to be painful and disruptive to athletic pursuits, although bone stress apparent on nuclear medicine bone scan and MRI is not consistently symptomatic [3,4]. Furthermore, pain severity is not well correlated with radiological severity [5]. Bones remodel in response to changes in type or intensity of chronic mechanical loading in order to adapt their density and morphology to best withstand future loads of the same nature (a phenomenon reflecting Wolff’s Law) [6]. It is this precise reason that exercise is beneficial for bone. In some cases, the adaptive process is insufficiently rapid to replace localised microdamage which, with further loading, can coalesce into one or more stress fractures. Furthermore, the process of remodelling and repair of microdamage involves an initial stage of resorption before new bone is formed [7]. It has been hypothesised that this transient period of increased porosity temporarily places bone at greater risk of further tissue damage under ongoing loading, thus creating a positive feedback cycle culminating in even greater risk of stress fracture [8]. The influence of adolescent growth on this cycle is not fully understood, but there is no evidence to suggest a different process occurs in the growing athlete. Microdamage and repair may manifest with degrees of upregulated metabolism (such as periostitis) and/or inflammation in or around the bone tissue (oedema) which may or may not be symptomatic or radiologically evident [3]. Attempts to categorise BSI severity clinically and radiologically have been only moderately successful [5]. This complexity, coupled with marked individual differences in pain perception and tolerance [9,10], accounts for the spectrum of bone tissue disturbances referred to as BSIs, the disparity in abilities to tolerate similar forms and intensities of training, and marked variation in times to recovery.

Athletes training in a sport involving dominant limb use such as tennis, high jump and long jump have long exhibited greater BMD (bone mineral density) or cross-sectional area in the dominant limb [11]. For exercise to stimulate bone adaptation, loading must be high-magnitude and/or applied rapidly. Examples include jumping, hopping and bounding as well as progressive resistance and power training, whereas swimming and cycling are relatively ineffective [12]. Bone also responds positively to unusual or novel forms of loading [13].

## 3. Epidemiology of Bone Stress Injuries in Adolescent Athletes

Lifetime prevalence of athletic stress fracture is reportedly 10% [14] but reports of incidence in adolescence range between 0.8 and 19% [15,16,17,18]. Teenagers may be more at risk of bone stress injury than young adults, with those aged 15–19 representing the largest proportion afflicted in an athletic population (42.6%) [19,20]. Additionally, recurrence rates may be up to 21% in collegiate sport, thus attention to management of risk factors in this age group is indicated [15].

### 3.1. Commonly Affected Sites

In athletes under the age of 20, 77% of stress fractures reportedly occur in the lower limb [21]. A description of 389 BSI occurring in high school athletes across a variety of sports, reported stress fractures occurring most commonly in the leg (40.3%), foot (34.9%), and lumbar spine or pelvis (15.2%), with upper limb and thoracic BSI being relatively uncommon (2.8%) [16]. It is possible this pattern of prevalence is a function of relative exposure and the sports represented, rather than a greater predisposition of the lower limb bones to BSI. Tibial BSI predominantly occur in sports that include repetitive jumping/landing (basketball and gymnastics) and running. Incidence of tibial BSIs in adolescent runners has been reported as 0.29/1000 AE (athletic exposures) for stress reactions and 0.06/1000 AE for stress fractures [22]. The most common location is the distal two thirds of the tibia, more specifically, at the junction of the mid to distal thirds [23], which corresponds to the narrowest cross section of the tibia and the likely site of greatest strain during loading [24]. In pre-professional dancers, BSI can represent up to 19% of injuries over an academic year, with tibial stress fractures taking the longest time to return to full dance [17]. When managed conservatively, tibial BSI may require 6–27 weeks to return to sport depending on severity [2,25,26].

### 3.2. Sports-Specific Bone Stress Injury

An overview of bones affected by BSI in childhood and adolescence is summarised in Table 1, along with the sports or activities commonly associated with them. Several studies have compared risk of BSI across different sports. While trends are evident, it is difficult to fully ascertain risk by sport as the gamut of sports compared, age of participants, and levels of training have been inconsistent between studies.

**Table 1:** Stress fracture locations in adolescent athletes, related sports, and associated activities.

Location	Examples of Sports	Additional Considerations
Tibia	Running (endurance and track) [18,22] Basketball, netball, volleyball [16,19] Gymnastics [27] Ballet [17] Figure Skating [28] Track and Field [16,19]	19.4% recurrence in collegiate athletes [15]. Include medial malleolus stress fracture or reaction in running or jumping athletes [29]
Metatarsal (Figure 1)	Running (athletics) [20] Gymnastics [27] Basketball [21,30] Volleyball [15] Ballet [17,31,32]	Most common bone affected in runners and highest rate of recurrence in collegiate athletes (29.2%) [15,18] Fifth metatarsal stress fractures have a high risk of delayed healing or non-union (Jones’ Fracture) [33] Pre-professional and young professional dancers experience more metatarsal stress fractures than senior professional dancers [31,32]. May reflect self-selection bias, i.e., injured dancers not progressing to professional level.
Tarsals (cuneiform, navicular, talus, calcaneus, cuboid)	Athletics [20] Basketball [16,21] Soccer (football) [16] Lacrosse [16] Figure skating [28] Ballet [17,29]	Consider tarsal coalition and bipartite navicular for differential diagnosis [34] Cuboid stress fractures are uncommon and may present similarly to an ankle sprain. CT diagnosis may be required [35] Comprise 13–19% of injuries in junior figure skaters [36]
Fibula	Running (track) [15] Soccer [15] Basketball [15] Figure skating [28]	9.7% prevalence in collegiate athletes [15]
Lumbar spine	Cricket [4,37,38] Tennis/Racquet sports [39] Gymnastics [15] Ballet [32]	Recurrence in 22.2% of collegiate athletes [15] Common presentation on opposite side to the dominant throwing arm or kicking leg and commonly, L5 vertebral level is affected [39] May span several vertebral levels in cricketers [38] Associated with repetitive lumbar extension with or without rotation for example, arabesques, gymnastic walk-overs, flips or pitching/bowling [4,32] Pars stress reactions and fractures are known to be among the top five most frequent paediatric sports injuries for both sexes [40]
Sesamoid	Running (endurance) [41] Soccer (football) Basketball and volleyball [19] Ballet [41]	Common in sports requiring weightbearing on an extended first toe. Possible association with pronation of the foot or hallux valgus [29,42]
Ribs	Rowing [43] Baseball/Pitching sports [19,44] Tennis [19] Swimming [45]	Reported 8–16% incidence during rowing career and associated with sudden increase in training load, poor rowing biomechanics or a change in rowing blade [19,43,46] First rib BSI may present as pain in the dominant posterior shoulder or upper thorax [44]
Olecranon and medial epicondyle	Baseball [19,47]	May occur following repetitive valgus stress forces and olecranon traction via the triceps tendon in pitching or throwing sports [19,47]
Pelvis/Sacrum	Running (endurance and athletics) [18] Football (Soccer) (kicking, sprinting or cutting) [19]	Both have a high proportion of trabecular bone, thus may be related to energy availability and menstrual regularity [48,49,50,51] Osteitis pubis (stress reactions at the pubic symphysis) often occur in adolescent and adult footballers [52]
Femur	Running (endurance and athletics) [15,53]	Coxa varum of the femur may be a contributing factor [53]
Wrist (Distal radius and carpal)	Diving [54] Tennis [55,56] Gymnastics [27]	Distal radial epiphyseal bone stress injuries may occur in children and adolescents yet to experience growth plate closure [57,58]
Patella	Gymnastics [59] Jumping sports (e.g., volleyball) [60]	Very rare [19] Consider bipartite patella as differential diagnosis [59]

Running as a mechanism of injury may account for up to 50.5% of BSI in athletes under 20 years of age [21]. Runners who have had prior involvement in ballet or gymnastics (aesthetic sports) have a higher risk of developing stress fractures than ball sports competitors, the reasons for which are not fully understood [18]. Aside from running (50.5%), in 222 cases, other stress fracture provoking activities include throwing (7.7%), jumping (6.7%), kicking (3.8%) and hitting a ball (3.4%) [21]. Many sports involve a combination of running and kicking, or running and jumping, thus differentiating one specific precipitating factor is challenging.

Ball sport athletes generally have higher than average bone mass, and individuals with a history of participation in ball sports as adolescents appear to be protected from stress fractures in future athletic activity [18,61]. However, playing ball sports involves considerable running and can therefore be associated with incidence of BSIs in adolescent athletes at the time of involvement [16]. Female high school athletes participating in ball sports such as soccer, volleyball and basketball appear to experience a higher incidence of BSI than their male counterparts, particularly in the lumbar spine and lower limb [16,21,62].

Incidence of BSI in aesthetic sports (10%) may be even higher than endurance sports (such as running; 8%), with prevalence in technical or ball sports being negligible by comparison [20]. The progression of gymnasts and figure skaters (aesthetic athletes) into elite level competition (Olympic level) during adolescence likely results in higher training loads at younger ages than other sports [63], which may partly account for the higher prevalence of BSI in these young athletes.

The risk of stress fracture in swimmers is not lower than those participating in impact sports [12] and may include upper extremity and rib BSIs. Although swimmers are unlikely to sustain lower extremity impact-related BSI while swimming [16], low BMD due to the weight-supported nature of the sport may render swimmers susceptible to BSI during lower limb-loading cross training activities [51].

Stress fractures at highest risk of poor or delayed healing include the anterior tibia, superior femoral neck, medial malleolus, talus, navicular, proximal fifth metatarsal, pelvis, hallux sesamoids and patella; likely as a result of high tensile loading and/or poor blood supply [29].

## 4. Risk Factors for Bone Stress Injuries in Adolescent Athletes

Sudden changes in training, low energy availability, race, previous stress fracture, bone mineral density (BMD), genetics, sex, biomechanics, hormone disruption, medications, and exercise history are broadly considered to be risk factors for BSI at any age [42,64,65]. The extent to which each factor contributes to the development of stress fractures in the young athlete is yet to be fully understood and likely differs from individual to individual [66].

### 4.1. Training Load and Early Specialisation

A change (type, intensity, surface, apparel) in training is the most common precursor to BSI, theoretically a function of the adaptive response to altered mechanical loading. A particularly intense period of increased or altered loading or inadequate rest predisposes to injury during the aforementioned window between the bone resorption and bone formation phases of bone remodelling [23,29]. A high number of weekly training hours may also place an athlete at increased risk of BSI by virtue of the accumulation of load related microdamage [20,67]. Training load describes the relationship between training volume (time) and training intensity (physiological demand) [68]. An increase in either volume or intensity will therefore affect risk of BSI [67].

As all athletes and sports are different, there is no known training threshold at which BSI are likely to occur. Of the female teenage athletes from The Japan Institute of Sport, those completing a higher number of training hours per week were most likely to sustain a stress fracture [20]. Others have shown that female adolescents exercising 12–16 h or more per week have an increased risk of BSI [30,67,69]. In one prospective study, 90% of stress fractures recorded occurred in adolescent females training for ≥1 h per day [70], and dancers training more than five hours a day appear to be at increased risk [71]. Male adolescents with a running mileage greater than 30 miles per week are at greater risk of stress fracture than non-running athletes or those with lower weekly mileage [65]. Although not evidence-based, one novel metric that has been proposed for adolescents is that risk of overall injury increases when the number of weekly training hours exceeds athlete age [63]. The exact relationship between number of weekly training hours and BSI risk will be highly individual, being dependent on personal anthropometrics and physical conditioning, as well as the nature of loading and training intensity. Undoubtedly it is an area in urgent need for quality research, which will be challenging due to the complexity of the individual considerations.

Early specialisation (common in gymnastics, diving, swimming, figure skating, and ballet) and engaging in year-round sport-specific training or playing in multiple teams of the same sport may predispose to injury along with other adverse health outcomes including burnout and sports discontinuation [63]. Injury risk is also higher for those involved in individual sports [63,72]. Involvement in a variety of sports, free physical play and/or cross training activities increases the variety of multidirectional osteogenic stimuli and development of a diverse range of motor skills [30,63,73] which may theoretically protect against BSI; however, direct evidence is yet to be reported.

### 4.2. Bone Mineral Density

Male adolescent runners were observed to have lower spine BMD if they also had a history of stress fracture, particularly at trabecular bone sites [49,65]. Low spine and whole-body BMD (Z-score ≤ −1.0) was found in 40% (*p* = 0.232) and 75% (*p* = 0.006) of female adolescent athletes with stress fracture respectively [20].

Female athletes with a history of stress fracture may have lower foot BMD than fracture-free athletes [14]; however, the coexistence of lower lean mass, leg-length discrepancy and menstrual disruption confounded the ability to make a direct connection between bone mass and BSI [64]. There is some evidence to suggest that for adolescents with stress fracture, the injured limb may have lower BMD than the non-injured limb [74]. The relationship of low bone mineral density to BSI as an isolated risk factor in adolescent athletes is not yet fully understood.

### 4.3. The Female Athlete, Menstruation and Hormones

Longitudinal studies investigating high school athletes report that the preponderance of stress fractures (63%) occur in girls and that female athletes experience higher grades of stress fracture than males [16,50,75]. A higher incidence of female BSI than male is prevalent across a wide variety of sports at the high school and collegiate levels [15,16]. Female sex per se is likely to be secondarily rather than primarily related to risk of BSI, by virtue of less robust bone size and lower muscle mass and strength [76].

Eumenorrheic athletes have a normal menstrual cycle, defined by 12 menstrual periods per year. Oligomenorrhea denotes irregular menstruation, or 6–9 menstrual periods per year. Amenorrhea is defined as a lack of menstruation for 3 months or more or alternatively <6 menstrual periods per calendar year [77]. Secondary amenorrhea occurs after at least one menstrual cycle has occurred (menarche), whereas primary amenorrhea is defined as never having menstruated. For female adolescents who have newly commenced menstruation (menarche), it may be difficult to ascertain menstrual dysfunction until some months have passed. Larger proportions of amenorrhoeic athletes than eumenorrheic athletes and non-athletes report a history of fracture (47%, 25.7% and 12.5% respectively) [78]. Decreased oestrogen in female athletes with menstrual disturbances appears to affect density of trabecular bone before cortical bone and thus susceptibility to trabecular site BSI may be increased [50].

Aside from sex hormone disturbances, cortisol may also be elevated in young adult females who are engaged in cognitive dietary restraint; however, elevated cortisol levels are likely a secondary function of low energy availability rather than a direct risk factor for bone stress and has not been widely studied in adolescent athletes [79,80]

It can be challenging for female athletes to achieve sufficient energy intake to establish and maintain regular menstrual cycles when training at very high loads. While the average age of menarche is 12.5–12.7 years [78,81], delayed menarche is common in adolescent athletes (age > 15 years), particularly in aesthetic or weight class sports such as gymnastics, cross country, rowing and dance [48,82]. For example, mean age of menarche recorded in a population of 147 adolescents with BSI was 16 and an association between amenorrhea and incidence of stress fracture was observed [20]. As BMD increases with gynaecological age (months since age of menarche) in non-athlete adolescents [51], delayed menarche may cause athletes to reach peak bone mass at a later chronological age [83], thereby placing them at increased risk of BSI in the intervening years.

The Female Athlete Triad (Triad) has been recognised for several decades and is currently defined as the interrelationship of low energy availability with or without disordered eating, menstrual dysfunction and low BMD, and exists on a spectrum of health to disease [77,84]. Relative Energy Deficiency in Sport (RED-S) is a newer term that includes the Triad and more broadly describes the effects of low energy availability on potential health and performance [85]. For females, low energy availability has been established as having a dietary intake of <30 kcal/kg fat free mass/day [86]. Emerging evidence suggests this may be lower for males (<29.5 kcal/kg fat free mass/day) [87]. Recognising signs of Triad/RED-S can be helpful when considering risk of BSI. The RED-S Clinical Assessment Tool (RED-S CAT), Low energy Availability in Females Questionnaire (LEAF-Q) and Triad Cumulative Risk Assessment (CRA) may identify disordered eating behaviours and gastrointestinal symptoms relating to menstrual disturbances and low energy availability. The Triad CRA take DXA measures into account when available, as well as menstrual history and dietary practices [77]. Using the Triad CRA, the risk of bone stress (low, medium or high) was determined [48]. When applying the Triad CRA in an adolescent population (<18 years), 85% ideal body weight for age should be used instead of the BMI cut-offs specified in the Triad CRA to determine risk category [77]. Modifications for the young female runner should be considered for girls who have not reached menarche but are less than 15 years of age, as menstrual status cannot be determined. A LEAF-Q score of >8 is classed as of high risk for reduced energy availability and scoring may also draw an athlete’s attention to the important relationships between menstrual regularity, gastrointestinal health and injury occurrence [88].

### 4.4. Male Athletes with Low Energy Availability

Boys with low energy availability may be harder to identify, given menstrual disturbance cannot be used as a marker of hormone status. Male athletes with low energy availability or high exercise dependence are likely to have low body weight and may experience elevated cortisol, low testosterone and hypogonadotropic hypogonadism associated with low BMD, thus increasing their risk of BSIs; however, further investigations are required in adolescents [65,89,90,91,92]. Sport-specific energy availability questionnaires such as the Dance Specific Energy Availability Questionnaire (DEAQ) for dancers and the Sport-Specific Energy Availability Questionnaire with Interview (SEAQ-I) have been used in male dancers and cyclists respectively to identify low energy availability; however, these are yet to be trialled in adolescent populations and correlated with adolescent BMD measures [93,94]

A modified Triad CRA may assist in identifying males at risk of bone stress injury as seen in a collegiate level population [90]. While this has not been thoroughly explored in male adolescents, evidence supporting relationships between low levels of sex hormones, low body weight and nutritional deficits (or disordered eating) exists in adult male athletes [65,89].

### 4.5. Dietary Considerations

Serum concentration of 25(OH)D3 in 1200 female Navy recruits aged 16–20 years (600 who developed stress fracture within 180 days of service and 600 who did not) revealed reduced chance of developing tibial or fibula stress fractures with higher serum 25(OH)D3; however, this was consistent only in Caucasian participants [95]. A daily combination supplement (calcium and vitamin D 2000 mg and 800 IU respectively), effected a 20% reduction in stress fractures in 3700 Navy recruits [96]. Daily supplementation of calcium (2000 mg) and vitamin D (1000 IU) over a 12-week period in new Marine Corps recruits (age range 16–21) improved vitamin D status and reduced markers of bone turnover, although effects of supplementation on BSI were only significant in the summer, suggesting sunlight exposure is perhaps a more potent and efficacious source of vitamin D [97].

Male adolescents who ingest less than one calcium-rich food per day in combination with low body weight and history of bone stress have an increased risk of BSI [65]. The practicality of achieving adequate calcium day to day may be a barrier for adolescent athletes. For example, females who followed a high-calcium diet for 2 years (dairy products) achieving 1160 mg daily had greater BMD than controls, but follow-up studies demonstrated that voluntarily achieving this intake was difficult [98]. Calcium serology was not found to differ between female teenage athletes who had experienced stress fractures and those without [20]; however, single serology may not be ideal method to monitor calcium status. While adolescent females who consumed ≥3 serves of calcium daily were not found to be at lower risk of stress fracture, for those participating in ≥1 h of daily exercise, vitamin-D intake was positively associated with lower fracture risk [70].

### 4.6. Eating Disorders

Adolescents with prior history of an eating disorder are up to five times more likely to develop a BSI [18]. Low energy availability should not be confused with a true eating disorder such as anorexia nervosa, which is a clinical eating disorder [78,99]; however, both states of energy deficit may result in hormonal disturbance and irregular or absent menstruation [100]. Suffering an eating disorder in adolescence increases the risk of poor bone health in later life, and short-term gains in weight (less than 6 months) do not benefit bone mineral accrual to the same extent as longer-term weight and hormone maintenance [101].

### 4.7. Sleep and Stress

Disturbed sleep patterns in the adolescent athlete due to training commitments, social habits, academic requirements and use of technological devices affect physiological recovery and thus may predispose to injury [102]; however, self-reported sleep and stress levels appear to be better in elite athletes than age- and sex-matched non-athletes [103]. However, general psychological stress and poor sleep was associated with BSI in adolescent girls [75] and adolescents who have less than eight hours of sleep per night over an extended period are more likely to experience general injury [104]. A large-scale military study found that introducing a minimum sleep regimen (6 h) reduced incidence of stress fractures in new Israeli recruits (minimum age 18) from 31% to 11% [105].

### 4.8. Biomechanical Factors

Certain foot types and postures have been associated with an increased risk of tibial stress injury, for example pes cavus (high foot arch) and pes planus (flat foot arch), leg length inequality, and increased hip internal range of motion and Q-Angle (hip-knee alignment) [22,64,76,106]. Adults with small bone morphology and narrower cortical width are at greater risk of tibial bone stress injury [64], but it is not known if a similar relationship exists in adolescent athletes.

### 4.9. Medications

There is no specific evidence linking any medication with BSI in adolescent athletes as no studies have been undertaken; however, there is considerable evidence that certain medications have adverse effects on bone and should therefore be considered to present a degree of risk for stress fracture. Medications or treatments that may have adverse effects on BMD include glucocorticoids, anticonvulsants, antidepressants, methotrexate, antiretrovirals and radiation therapy [107].

#### 4.9.1. Asthma

The effects of inhaled corticosteroids for conditions such as asthma on BMD, growth and development in childhood have been the subject of considerable discourse [108,109,110,111]. The potency of such medication, from greatest to least commonly administered, is fluticasone propionate, beclomethasone dipropionate, budesonide and triamcinolone acetonide [110]. Corticosteroids inhibit linear growth through downregulation of growth hormone and inhibition of osteoblast activity [110]. Bone growth is affected by glucocorticoids through inhibition of absorption of calcium from the intestine, increased excretion and promotion of bone resorption. Despite glucocorticoids being recognised as a contributing factor to low BMD, common inhaled corticosteroid (ICS) asthma medication appears to have no significant impact on bone [108,109]. There are insufficient longitudinal studies of adequate length investigating the effects of glucocorticoids on BMD or BSI, with many observing for only 12–13 months (or less). Asthmatics generally use the medication for much longer periods during childhood [109,111]. A 24-month prospective observational study reported greater BMD accrual in those treated with steroidal (fluticasone) than non-steroidal (nedocromil sodium) asthma medication; however, higher physical activity levels in the fluticasone group confounded results [112].

#### 4.9.2. Dermatological Conditions

Adolescents may be prescribed medication for skin conditions such as acne; for example, vitamin A-derived isotretinoin (Roaccutane). Clinical trials have shown that BMD is not adversely affected by 6 months of isotretinoin therapy [113]; however, longer term use may pose different risks that may affect bone health. For example, vitamin D deficiency can occur due to high doses of vitamin A in the drug and the recommendation to limit sun exposure when using the drug [114]. Low-dose oestrogen oral contraceptive pills, sometimes prescribed to female adolescents to manage acne, may inhibit normal BMD accrual during adolescence [78,115].

### 4.10. Other Risk Factors

#### 4.10.1. History of Fracture

Prior history of stress fracture is positively related to risk of future stress fracture likely a function of the intrinsic characteristic(s) that predisposed to the first, such as low BMD, low trabecular density and bone strength [16,18,20,65,74]. For female adolescent athletes, those who have had a prior stress fracture may be 8.39 times more likely to sustaining a subsequent stress fracture [20]. One epidemiological study involving high school athletes involving both male and female athletes found 18.1% of 389 bone stress injuries sustained over 8 years were recurrent [16]. Additionally, the risk of BSI may double in females with a family history of stress fracture compared to those with no family history [30].

#### 4.10.2. Race

Lower DXA scores and history of previous fracture was associated with increased risk of fracture in Caucasian adolescent females when compared to other races; however, those findings did not differentiate between traumatic fractures and stress fractures [116]. The observation is; however, consistent with findings from military personnel. Of 600 Navy recruits, Caucasian females (≤20 years) accounted for 54.4% of stress fractures in basic military training [95]. In a larger population of mixed defence personnel (<20 years), those of Caucasian race were found to have a higher incidence of stress fracture than Blacks [117]. The observation that African American adolescent females have higher BMD than females of other races [83] may, at least in part, account for their lack of predisposition to BSI.

#### 4.10.3. Bone Turnover Markers

Several military studies involving new recruits under 20 years of age found bone turnover markers did not predict incidence of bone stress injury [118,119,120], which is in keeping with similar findings in young adult athletes [121].

#### 4.10.4. Joint Hypermobility

Joint hypermobility syndrome (but not generalised joint hypermobility) has been associated with increased prevalence of bone stress injury in collegiate athletes with a Beighton score of ≥4/9, joint pain in four or more joints for ≥3 months, history of joint dislocation and skin elasticity [122]. Generalised joint hypermobility classification in this instance was described as having combined signs of Beighton score ≥5/9, anxiety and fatigue. There is a paucity of quality research investigating the relationship between bone stress injury and hypermobility, particularly in adolescents.

## 5. Diagnosis

### 5.1. History and Symptoms

Usual patient presentation reveals a history of gradual onset pain in a bony location, symptomatic initially with impact activities [66]. Most will report some form of change in training in the weeks immediately prior to the development of the discomfort.

Pain is usually felt during the offending activity, particularly in the initial phase of impact loading (running, jumping, pitching, compression in weightbearing of the carpals, arching the spine into extension). The pain may be diffuse for lower severity BSI but tend to become highly focal once a stress fracture has developed. More than one BSI may be present in a single bone. Palpation of a superficial BSI will be painful, as will a tap test or percussion test using a finger or a reflex hammer on the affected site. For lower limb BSI, jumping, hopping, or rising on to the tip toes may reproduce the pain; however, caution is advised attempting impact activities with high grade BSI to avoid worsening the injury [26]. Once advanced, pain may be present with walking, standing or even at rest (night pain). Non-muscular low back pain that has been present for greater than seven days in athletes of high-risk sports (cricket, gymnastics), should be investigated for a lumbar pars bone stress reaction, especially if the pain is unilateral and worse with spine extension [123]. Pain tolerance is highly variable and is a poor index of BSI severity and time to physiological healing [5].

### 5.2. Clinical Signs

At superficial sites such as the anterior tibia, tarsals or metatarsals, the area will likely be tender, potentially inflamed, red and/or warm with palpation [2]. A painful lump over the anterior tibia may be present.

Certain tests can be performed for positive signs. Femoral neck stress fracture pain may be felt at the hip or referred to the knee. Clinical signs include a positive ‘fulcrum test’ [124] (where the patient’s femur is positioned halfway off the edge of the bed and stabilised proximally as pressure is applied distally) or hop [29]. Important differential diagnosis for a paediatric or adolescent population may include slipped capital femoral epiphysis (SCFE), osteoid osteoma, osteosarcoma or round cell lesions, which usually require surgical management [125]. Diagnosis is usually confirmed with MRI [53]. Lumbar pars stress reactions will be painful during active lumbar extension or a combination of lumbar extension and spinal rotation towards the side of pathology. Pelvic stress fractures (osteitis pubis) will be painful with adductor activation “squeeze test” or resisted flexion, abduction and external rotation (FABER) [52]. In the case of suspected rib fracture, compression of the thoracic wall or percussion testing may reproduce pain during physical examination [43].

### 5.3. Imaging

Imaging is often required to confirm diagnosis of a BSI and may be indicated following positive clinical signs. The choice of imaging may be based on cost and availability to the athlete, as well as prior radiation exposure. Multiple types of imaging may be required to either confirm or negate diagnosis; however, radiation exposure should be particularly minimised in adolescent athletes. Importantly, the determination of severity can be highly inconsistent between different imaging modalities (plain films, CT, MRI and nuclear medicine) [5].

#### 5.3.1. Magnetic Resonance Imaging 

The early identification of oedema and lack of radiation makes MRI the favourable imaging modality for the adolescent athlete, but it is costly by comparison with nuclear medicine bone scan. MRI is appealing for early diagnosis of BSI due to high sensitivity and specificity, and differentiation from other tissue pathology. In terms of diagnostic accuracy, sensitivity of MRI has been recorded as 71.4–88%, specificity at 85.7–100%, accuracy at 78.6–90% with higher values for the tibia at one month of symptom onset [126,127]. Of all the imaging modalities, severity of tibial stress fracture from MRI is most likely to be associated with time to healing [5] and return to activity [50].

Progressive MRI screening in adolescent cricketers detected that 67% of athletes experienced some degree of bone oedema in the lumbar spine during a single season [128]. While a proportion of these lesions may be asymptomatic and scope for this type of novel screening may be limited due to time and cost in practice (particularly in developing countries), they may be helpful for prevention where circumstances permit.

#### 5.3.2. Bone Scintigraphy

Bone scintigraphy or a nuclear medicine bone scan involves injecting a radioactive material into the bloodstream in order to detect gamma rays given off by the areas of high uptake that occur at sites of increased bone metabolism [129]. Bone scintigraphy is recognised as a highly sensitive modality for diagnosis of BSI [130,131]. Diagnostic accuracy has been reported as 74–92.9% sensitivity, 73.8% specificity and 83.3% accuracy [129]. Nevertheless, use of a radioisotope and relatively high levels of ionising radiation is a consideration when choosing an imaging modality for adolescent athletes, and this has largely fallen out of favour, preferring the use of non-ionising radiation sources of magnetic resonance imaging (MRI).

#### 5.3.3. Plain X-ray

Plain radiography is not a sensitive diagnostic imaging modality in the acute phase of a BSI. Within 3 weeks of injury, a periosteal reaction may be evident on X-ray, but a stress fracture may never be detected beyond the presence of a callous [132].

#### 5.3.4. Computed Tomography (CT)

CT has high specificity for stress fracture but relatively low sensitivity (42%) and accuracy (52%) [127]. The relatively high ionising radiation thus suggests CT is not the ideal imaging modality for BSI in adolescents. CT is often considered to detect spondylolysis or pars BSI; however, MRI with thin cut protocol can be effective for detecting this injury without involving the application of high ionising radiation to the pelvis.

#### 5.3.5. Therapeutic Ultrasound

Therapeutic ultrasound has been investigated as a method for assisting diagnosis of tibial stress injuries in adults and may be a suitable modality for children if sufficiently sensitive. This is achieved by eliciting pain when placing the active sound head over the suspected area of bone stress and may identify periosteal irritation in the early stages. Pooled analysis of diagnostic accuracy of therapeutic ultrasound in a systematic review has been calculated 64% sensitivity and 63% specificity [133]. This method of diagnosis requires further validation, especially in paediatric and adolescent populations, and should not be solely relied upon in a clinical setting [134,135].

#### 5.3.6. Diagnostic Ultrasound

Diagnostic ultrasound, being cost effective, portable and radiation free, has undergone preliminary investigation as a means to diagnose metatarsal, tibial and rib stress injuries. Diagnostic ultrasound produces an image of internal tissues which may be indicative of hypervascularity in acute stages or callous formation in subacute phases of bone stress [135]. As this method cannot yet determine classification of severity of BSI, nor determine the presence of stress injuries in trabecular areas, further research is required regarding this potential of the imaging modality to diagnose BSI [135].

## 6. Management and Rehabilitation

There has been little to no work specific to management strategies for adolescent athletes with BSI; therefore, the following summary is largely an extrapolation from adult populations. Consideration for the growing skeleton should be factored into healing status from a BSI.

### 6.1. Conservative Management and Prognostication

Full or even partial participation in sports with a bone stress injury is difficult, especially as the usual cause of injury is a repetitive mechanism associated with the sport. Bone healing and rehabilitation may take several weeks, or months of full or modified rest. For this reason, BSIs, particularly stress fractures, can be associated with large time losses to participation [17,37,136]. Attention should be paid to identifying and modifying physical- or lifestyle-related risk factors predisposing to the BSI in order to avoid reinjury after return to full participation.

In general, treatment of the more severe lower extremity BSIs may involve non-weightbearing, crutches, splints, air casts or ‘moonboots’ (CAM walker boots) [21]. For the lumbar spine, a brace may reduce pain during activities of daily living; however, efficacy is yet to be confirmed [39,137]. Time to return to unrestricted sport for any BSI in collegiate level athletics is reportedly 12–13 weeks on average, depending on the grade and severity, yet may range from 6 to 30 weeks [25,50], and is highly dependent on the location of injury.

Providing a prognosis for athletes, their families and coaches is an ongoing clinical challenge. Patient symptoms, clinical exam and function are key indicators determining time to return to play. Systems for grading imaging, such as the Kaeding-Miller stress fracture classification system, may be useful for patient education and prognosis based on imaging results [138], or the Fredericson system can be used to classify tibial stress fracture severity from MRI into four subcategories (Table 2) [2]. The Torg (1, 2 and 3) classification system is used for proximal 5th metatarsal stress fractures [33]. Lumbar pars stress injuries are classified based on severity from bone stress reaction to pars defect and forward slippage of the vertebrae [39,139]. It is important to consider the length of time it has taken the athlete to report pain and seek treatment, as this may vastly affect time to healing [26,136]. Many pars stress fractures resolve conservatively (81%); however, if symptoms persist beyond six months, surgery should be considered for grade 2 or higher due to risk or presence of a forward slippage of the vertebrae (spondylolisthesis) [39].

While an injured athlete is required to rest from sport, stationary cycling, hydrotherapy and swimming, anti-gravity treadmills, Pilates (such as reformers and trapeze tables/Cadillac) and seated exercise can be tolerated pain free [26,140,141]. Such programs are frequently employed clinically [39,106,136] to maintain fitness but are unlikely to enhance healing of the BSI. Providing a means of alternate, lower load exercise will minimise immobilisation-induced muscle wasting and reduce the physical challenge of transitioning to return to sport. Some low-grade (grade 1) stress reactions can be managed with rest from aggravating activity and prophylactic support (bracing). Higher grades of BSI (grade 3 and higher), stress fracture or those involving the growth plate may require immobilisation and rest from the provoking sport for four to six months [57].

A modest return to normal training should be guided with gradual introduction of the aggravating activity (for example, running, throwing, spine extension) and simultaneous muscle strengthening over several weeks. Any recurring bone pain should be managed with 1–2 days rest [43,106].

#### 6.1.1. Low-Intensity Pulsed Ultrasound (LIPUS)

There is limited evidence to support the use of LIPUS in accelerating healing of BSI in adolescent athletes. Reviews have found LIPUS does not significantly reduce time to weight bearing, pain at 4–6 weeks, or radiologic healing for lower limb stress fractures [142]. By contrast, earlier return to sport has been demonstrated in adolescents with lumbar pars stress reaction using LIPUS despite not achieving the recommended daily dosage of 20 min [136,143]. Effectiveness in specific child and adolescent populations across a variety of body regions is yet to be investigated.

#### 6.1.2. Electric Field Stimulation

A randomised controlled trial of a wearable electrical field stimulator observed reduced healing times only in the treatment group of adult participants with the most severe tibial stress injuries (Fredericson Grades 3 and 4) [144]. Further research investigating effects of electric field stimulation treatment in adolescents is warranted.

### 6.2. Surgical Management

In some cases, such as delayed or non-union, or widening of the fracture line, grafting or surgical fixation of high-risk stress fractures (e.g., anterior tibia) is required to progress healing [42]. Surgery is also often indicated for sesamoid stress fractures, which may involve fixation or sesamoidectomy; however, few data are available for children and adolescents [29,42]. Surgery for BSI of the elbow region from throwing sports may involve cannulated screw insertion or tension banding [145]. In adolescents who are still expected to grow, surgery may involve sliding pins, modified cannulated screws or planned surgical revision [125].

### 6.3. Other Management Considerations

#### Psychology in Rehabilitation

The occurrence of a BSI may be the first injury requiring an extended break from training and competing for an athlete. Adolescents may have limited experience of serious injury and the length of time required to rehabilitate can be intensely frustrating for them. As a result, many young athletes (especially those in pursuit of professional status) will attempt to continue to train through their BSI [21,146] which will likely compound the problem. Any psychological factors that may have contributed to the injury (compulsive exercise behaviours or disordered eating) will remain evident and potentially compromise the healing process. For this reason, a consult with a clinical psychologist may be helpful [77,147]. Fear avoidance and pain catastrophising are associated with athlete-reported decreased physical function when returning from injury [10]. Positive psychological support during injury may encourage young athletes to develop positive skills in rehabilitation that can be transferred into future training, such as learning coping, goal-setting, adapting or changing pain perception and understanding prevention [146].

## 7. Prevention of Stress Fractures in Adolescent Athletes

### 7.1. Recognising Stages of Growth

Bone density temporarily declines during periods of rapid growth during childhood. Thus, adolescents may be at increased risk of BSI at certain stages when growth is particularly rapid due to delays in bone mineralisation [57]. Stages of growth are difficult to predict but several strategies are available, including Tanner Staging, Peak Height Velocity (PHV) calculations, and radiography of the hand and wrist [148].

Age of Peak Height Velocity (APHV) provides a simple method to estimate of stage of the adolescent skeletal growth spurt based on standing and sitting height. It can be highly asynchronous with chronological age owing to individual differences in timing of growth [149]. Mean age at peak height velocity is approximately 12 years in girls and 13 years in boys [81,150,151]. After ages 13–14, following peak height velocity, bone strength typically increases and differences between males and females become more pronounced [152]. Bone loading during the phase of peak height velocity will theoretically optimise bone accrual [153], but there is an unpredictable and highly individual tipping point between osteogenic loading and loading that increases the risk of BSI. The optimal timing to load bone for injury prevention and the time during growth when adolescents may be more susceptible to BSI with high training volume have not been well substantiated.

### 7.2. Training Load

Once an athlete has been cleared to return to sport, training should begin at an intensity considerably lower than that at the time of injury. Increases in training intensity and variety should be gradual, with consistent monitoring for a return of symptoms.

Training load (volume) can be modified either by reducing training hours (duration) or reducing intensity of training, or both. Borg’s Rating of perceived exertion (RPE) is a subjective, individualised rating of intensity that can be a useful benchmarking index for adolescent athletes across a variety of sports and training scenarios; however, there is limited evidence to associate training load monitoring or planning in this way with reduction in BSI [68,154].

Differentiation between the protective effect of osteogenic exercise vs. over exercising and over training may be useful, especially in the context of an adolescent athlete with intrinsic risk factors for BSI. Novel loading or cross training may be beneficial for those who have been participating in their sport through childhood and adolescence. For example, a triathlete could benefit from a reduction in pool time and adding resistance-based cross training or land-based ball sports to stimulate bone development [58,63,73].

### 7.3. Resistance and Impact Training

Supplementary resistance and impact training for adolescents may protect against BSI and is encouraged at all stages of life to either maximise peak bone mass or slow its decline in later years [13]. Contrary to the suggestion that early resistance training may disrupt growth plate development and cause premature closure [155], a 2014 Consensus Statement [156] stipulates supervised resistance training should be encouraged in children and adolescents to promote bone development. In particular, multi-joint, high-intensity, impact and resistance exercise is favourable, for example, jumping [157,158,159].

Resistance or plyometric training for osteogenic benefit has been investigated in the adolescent female population over 6 months with strength gains achieved but no significant BMD changes [160]; however, 6 months is arguably an insufficient duration to detect changes in bone. Others have observed positive changes to BMD over longer training periods (9–15 months) of jumping and resistance exercise [161,162,163,164,165]. Additional benefits of ongoing resistance training over multiple years or sporting seasons have not been explicitly investigated, yet it has been observed that 58% of high school athletes who sustained BSI over a two-year period did not partake in weight training, which may suggest a protective effect [75]. Results from a study of the Brazilian martial art Capoeira plus jumping in pre-pubertal children revealed a positive effect on BMD, and one year after withdrawal those differences between exercising and non-exercising groups remained [166,167]. Those findings suggest osteogenic loading can benefit bone in childhood and potentially continue through to adolescence. Meta-analysis of strength training interventions in athletes as young as 12 years old demonstrated that a 10% increase in strength training volume is effective to reduce risk of overuse injury by 4% [168]. Adolescent male runners who also participate in plyometric training in season have been known to be at an increased risk of bone stress injury as opposed to those who did not; however, this was not the case for those who participated in weight training [18].

Muscle strength may have a protective effect against low BMD in females despite low energy availability, a high number of training hours and hormonal imbalances [169]. A skeletal loading program in conjunction with dietary intervention has shown to be effective in improving lumbar spine BMD in male adult cyclists with low energy availability [170]. As runners with reduced calf girth were more susceptible to stress fractures, building muscle mass may enhance BMD and reduce incidence of stress fractures [14].

Off-season or pre-season strength training may reduce the risk of overloading athletes as young as 12 years in periods of greater demand, as well as allow for supervision for correct technique, individual load progression and implementation of recovery time [168].

### 7.4. Diet

#### 7.4.1. Energy Availability

The Female Athlete Triad and Relative Energy Deficit in Sport (RED-S) can negatively influence hormonal cycles and disturb several other physiological processes such as immune function, mood and psyche, protein synthesis and cardiovascular health [50,77,78,85,171,172].

Coaches and athletic trainers who work with collegiate athletes may be aware of Female Athlete Triad and RED-S but may not always have a complete understanding of energy availability [173]. Furthermore, some male coaches may not be aware of female athlete triad [173]. Education for coaches and those working with young athletes at both elite and recreational levels may lead to earlier identification of athletes at risk of BSI and more cohesive long-term management [173]. Athletes of both sexes with suspected eating disorders require formal clinical diagnosis and planning of multi-disciplinary management including a dietitian, psychologist and sports/medical physician [99]. Participation in sports may require restriction if BMI falls below 16.5 [77,99].

#### 7.4.2. Calcium and Vitamin D Supplementation

Vitamin D is required to absorb calcium from the gut and is also active in the kidney to resorb calcium [174]. When serum calcium levels fall because of insufficient intake or excessive loss of calcium, 1,25(OH)2D3 (vitamin D) and parathyroid hormone production is increased to stimulate bone resorption, liberating calcium from the skeleton to restore serum calcium concentration. Long term, this form of calcium depletion will reduce bone mass and strength, therefore ensuring adequate dietary calcium is an intuitive strategy to prevent BSI. The efficacy of calcium and vitamin D intake and supplementation to reduce BSI is has not been established beyond observation studies that have determined associations rather than causation [18,75].

For adolescents (ages 9–18), 1100–1300 mg calcium is recommended daily, preferably from food sources [175]. If supplementation is required, optimal calcium absorption occurs with no more than 500 mg at once and a total of 2500 mg can be absorbed per day. Calcium citrate is the preferred form [176]. Vitamin D insufficiency range (serology) is 20–30 ng/mL, deficiency is <20 ng/mL, and athletes (not specifically adolescents) may benefit from 40 ng/mL [95,174]. Sun exposure may help produce endogenous vitamin D production, but safe sun exposure to optimise vitamin D level while reducing risk for skin cancer has not been established.

### 7.5. Biomechanical Screening

Biomechanical screening may identify the presence of risk factors for BSI such that preventative strategies could be put in place; for example, altering lower extremity kinematics and sporting technique (e.g., bowling, throwing, rowing) [145,177]. Regular musculoskeletal screening throughout training seasons has been recommended to identify developing BSI in order to implement timely activity modification or withdrawal from training [4]. Screening may also provide an opportunity to introduce strengthening programs; however, quality evidence from intervention trials is not yet available [178].

### 7.6. Medication

Addressing endocrine dysfunction is a challenge for the adolescent female athlete. For a female athlete prone to BSI with low BMD and amenorrhea or oligomenorrhea for whom the demands of training leading into performance and competition cannot be modified, oestradiol patches are an emerging therapy [179,180]. The aim of this intervention is to improve BMD through oestrogen replacement however, more research is required. Oral contraceptives do not restore BMD in athletes and in some cases, are detrimental to BMD [115,181,182,183]. Irrespective of oestrogen supplementation in the adolescent athlete, potential underlying causes of amenorrhea (energy availability, pituitary issues, ovarian issues, thyroid) should be addressed [180].

Medications typically administered to older persons with osteopenia or osteoporosis (bisphosphonates) are in most cases not appropriate for adolescent athletes, particularly females, due to their potential teratogenic effects and long-half life in bone [184]. Although case studies have evaluated intravenous bisphosphonates for young adult athletes prone to stress fracture, RCTs investigating the effectiveness and safety of this method of treatment in adolescent athletes or Female Athlete Triad are lacking [106,181]. Bisphosphonates (risedronate) did not prevent stress fracture in Israeli army recruits (mean age 19.1 ± 1.2) [185]. For the athlete with asthma on long term steroids, supplementation with calcium and vitamin D is advisable [186] but may be insufficient to fully protect from bone loss. Second-line agents including PTH analogues (Forteo) or other agents have not been adequately studied.

## 8. Conclusions

There is some evidence that adolescent athletes are at greater risk of bone stress injuries (including stress fracture) than their adult counterparts; however, minimal empirical data are available specific to paediatric risk factors, diagnosis, management and prevention. In the absence of such evidence, we have extrapolated from the adult condition and from what is known about strategies to optimise paediatric bone health. Undoubtedly, the gold standard approach to managing BSIs in adolescent athletes is prevention, and therein, several modifiable factors can be addressed to reduce risk. A multidisciplinary approach should include: enhancing bone development with targeted bone loading throughout childhood (maximising variety of loading and using resistance and impact training), managing training load with graduated increments in intensity and rest periods, optimising energy balance, calcium and vitamin D, rectifying biomechanical and kinematic anomalies, encouraging diversity of sports participation (minimising sports specialisation), and recognising and managing medications of relevance to bone health. Additionally, enhancing awareness of coaches and athletes on issues such as relative energy deficit in sport, and the benefits of diversity of training and psychological counselling will maximise an adolescent athlete’s ability to avoid or effectively rehabilitate from bone stress injury.

## Figures and Tables

**Figure 1:**
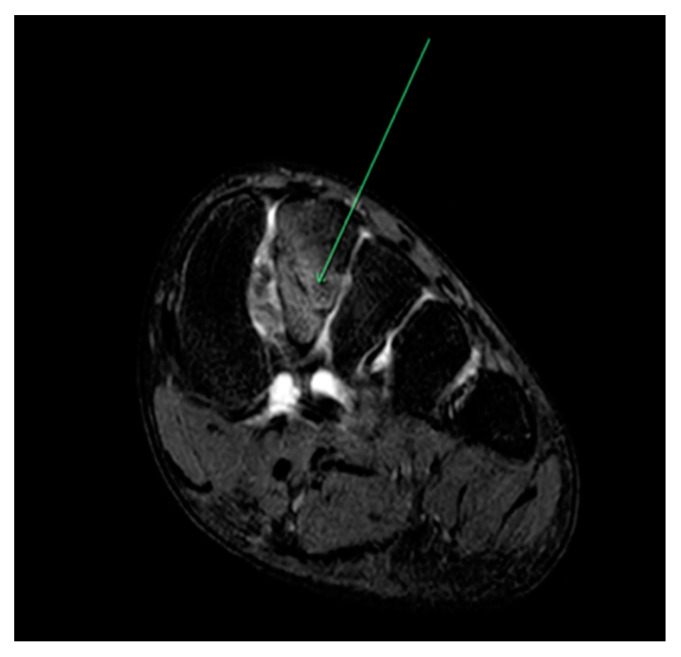
Coronal plane MRI of T2 weighted image of 2nd metatarsal stress fracture (as indicated by the green arrow) in 14-year-old ballet student training 25–30 h per week.

**Table 2:** Tibial Stress Injury (TSI) Image Grading Criteria (This table was published *Radiology*, 263, Beck BR, Bergman AG, Miner M, Arendt EA, Klevansky AB, Matheson GO, Norling TL, Marcus R: Tibial stress injury: Relationship of radiographic, nuclear medicine bone scanning, MR imaging, and CT Severity grades to clinical severity and time to healing, 811–818, Copyright Elsevier, 2012. [5]).

Grade	Radiography [132]	NM Bone Scanning [131]	MR Imaging [2]	CT Scanning [127]
0	No abnormality	No abnormality	No abnormality	No abnormality
I	Gray cortex sign; margin is indistinct, density lower	Linear increased activity in cortical region	Mild to moderate periosteal oedema	Soft tissue mass adjacent to periosteal surface
II	Acute periosteal reaction, density differs from rest of cortex showing incomplete mineralisation	Small focal region of increased activity	Periosteal oedema and bone marrow edema only on T2 weighted images	Increased attenuation of yellow marrow
III	Lucent areas in cortex, ill- defined foci at site of pain	-	Marrow oedema on T1- and T2-weighted images with or without periosteal oedema on T1- or T2- weighted images and loss of cortical signal void, intracortical increased intensity and intracortical linear hyperintensity	Increased hypoattenuation (osteopenia), intracortical hypoattenuation (resorption cavity), and subtle intracortical linear hypoattenuation (striation)
IV	Fracture line present	Very large focal region of highly increased activity	Low-signal-intensity fracture line with all sequences, moderate to severe periosteal oedema on T1- and T-2 weighted images, marrow oedema on T1- and T2- weighted images, may also show severe periosteal and moderate muscle oedema	Hypoattenuating line

## Data Availability

Data sharing is not applicable to this article.
